# Role of YAP/TAZ in Cell Lineage Fate Determination and Related Signaling Pathways

**DOI:** 10.3389/fcell.2020.00735

**Published:** 2020-07-30

**Authors:** Boon C. Heng, Xuehui Zhang, Dominique Aubel, Yunyang Bai, Xiaochan Li, Yan Wei, Martin Fussenegger, Xuliang Deng

**Affiliations:** ^1^Central Laboratory, Peking University School and Hospital of Stomatology, Beijing, China; ^2^Faculty of Science and Technology, Sunway University, Subang Jaya, Malaysia; ^3^Department of Dental Materials & Dental Medical Devices Testing Center, Peking University School and Hospital of Stomatology, Beijing, China; ^4^National Engineering Laboratory for Digital and Material Technology of Stomatology, NMPA Key Laboratory for Dental Materials, Beijing Laboratory of Biomedical Materials, Peking University School and Hospital of Stomatology, Beijing, China; ^5^IUTA Department Genie Biologique, Universite Claude Bernard Lyon 1, Villeurbanne, France; ^6^Department of Geriatric Dentistry, Peking University School and Hospital of Stomatology, Beijing, China; ^7^Department of Biosystems Science and Engineering, ETH-Zürich, Basel, Switzerland

**Keywords:** differentiation, Hippo, signaling, stem cells, TAZ, YAP

## Abstract

The penultimate effectors of the Hippo signaling pathways YAP and TAZ, are transcriptional co-activator proteins that play key roles in many diverse biological processes, ranging from cell proliferation, tumorigenesis, mechanosensing and cell lineage fate determination, to wound healing and regeneration. In this review, we discuss the regulatory mechanisms by which YAP/TAZ control stem/progenitor cell differentiation into the various major lineages that are of interest to tissue engineering and regenerative medicine applications. Of particular interest is the key role of YAP/TAZ in maintaining the delicate balance between quiescence, self-renewal, proliferation and differentiation of endogenous adult stem cells within various tissues/organs during early development, normal homeostasis and regeneration/healing. Finally, we will consider how increasing knowledge of YAP/TAZ signaling might influence the trajectory of future progress in regenerative medicine.

## Introduction

**FIGURE 1:**
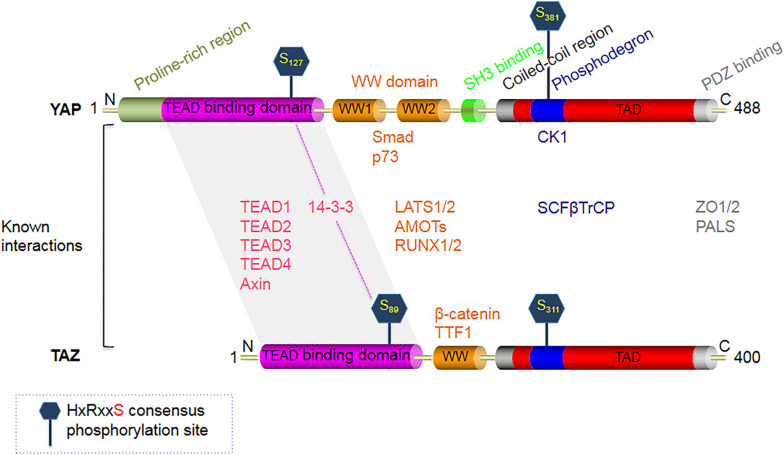
Structural domains of the Hippo signaling pathway effector protein YAP (65 KDa), and its smaller paralog TAZ (43 KDa). YAP is constituted of an N-terminal proline-rich region, followed by a TEAD-binding region, two WW domains, a Src homology domain 3 (SH3) binding motif, a coiled-coil domain (CC), a phosphodegron motif, a transcription activation domain (TAD), and finally a C-terminal PDZ-binding motif. TAZ is similar in structure to YAP, except that it lacks 3 domains present in YAP - the N-terminal proline-rich region, one WW domain, and the SH3 binding motif. The major phosphorylation target sites of LATS1/2 on YAP are S127 and S381, while the corresponding target phosphorylation sites on TAZ are S89 and S311. Upon phosphorylation, YAP/TAZ binds to 14-3-3, which inhibits their translocation into the cell nuclei.

**FIGURE 2:**
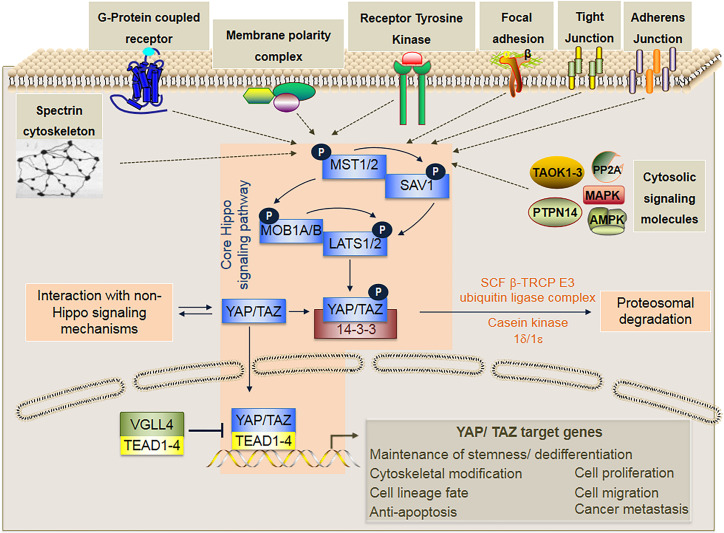
In mammals, the core Hippo signaling pathway is a kinase cascade composed of MST1/2, LATS1/2, SAV1, MOB1A/B, YAP/TAZ, the 14-3-3 protein that binds only to phosphorylated YAP/TAZ, and the TEAD transcription factors (TEAD1-4) that activates transcription of specific target genes upon binding to unphosphorylated YAP/TAZ. Activation of the core Hippo signaling cascade occurs through phosphorylation of either MST1/2 or LATS1/2 by various upstream signaling mechanisms that may involve various cytosolic signaling molecules (i.e., PP2A, TAOK1/2/3, MAPK, AMPK, and PTPN14), Adherens junctions (AJs), Tight junctions (TJs), Focal adhesions (FAs), Receptor tyrosine kinases (RTKs), Membrane polarity complexes (i.e., Crumbs, Scribble, aPKC-PAR) G-protein coupled receptors (GPCRs), and Spectrin cytoskeleton. Phosphorylation of YAP/TAZ inhibits nuclear translocation, and ultimately leads to proteasomal degradation via further phosphorylation of YAP/TAZ by casein kinase 1δ/1ε and ubiquitination by the SCF β-TRCP E3 ubiquitin ligase. By contrast, when the core Hippo signaling cascade is not activated, YAP/TAZ remain unphosphorylated and are translocated to the cell nuclei where they bind to TEAD transcription factors (TEAD1-4) and subsequently activate transcription of specific target genes that are involved in diverse cellular functions. Within the cell nuclei, VGLL4 can repress target gene expression by acting as a competitive inhibitor of YAP/TAZ binding to TEAD transcription factors.

YAP (Yes-associated protein, also known as YAP1) and TAZ (transcriptional co-activator with PDZ-binding motif) are two homologous transcriptional co-activator proteins (Webb et al., 2011) (Figure 1), which shuttle between the cytosol (phosphorylated inactive state) and cell nuclei (unphosphorylated active state) to regulate target gene expression via binding interactions with TEAD (TEA/ATTS domain) transcription factors (Lin et al., 2017b). Signaling mechanisms regulating YAP/TAZ activity can be broadly divided into two categories; that which (i) interact directly and is dependent on the canonical Hippo signaling pathway (Figure 2), and that which (ii) operate independently of it (Pocaterra et al., 2020).

**FIGURE 3:**
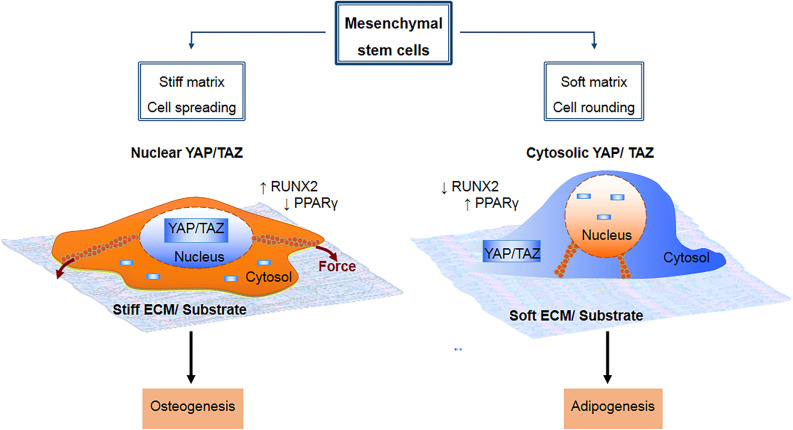
Mesenchymal stem cell lineage fate is known to be influenced by mechanical cue-induced localization and activation of YAP/TAZ. On a stiff substrate, there is increased integrin clustering and formation of focal adhesions, which in turn enhances F-actin polymerization and formation of stress fibers. This cause the cell to spread out over a larger surface area due to increased torsional forces within the stress fibers, and promotes cytosolic to nuclear translocation of YAP/TAZ, which drives osteogenesis by upregulating RUNX2 and downregulating PPARγ. By contrast, a soft substrate is non-conducive to formation of focal adhesions and stress fibers, causing the cell to adopt a more rounded morphology with less spreading area. This in turn promotes sequestration of YAP/TAZ within the cytosol, which drives adipogenesis by upregulating PPARγ and downregulating RUNX2.

**FIGURE 4:**
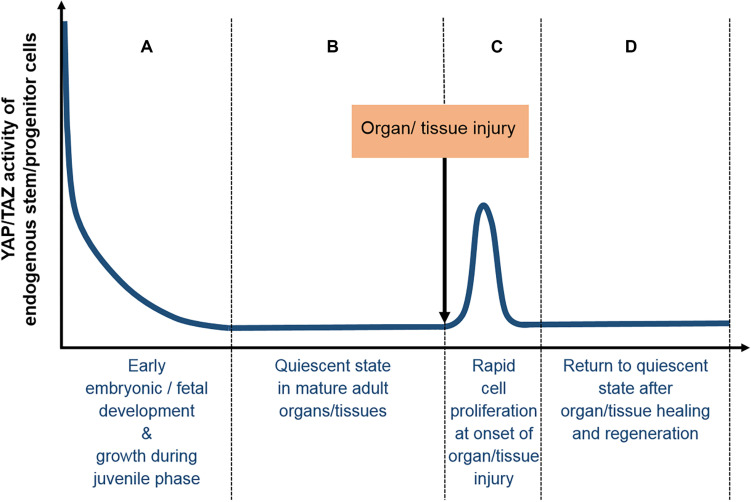
Typical YAP/TAZ expression profile in tissues/organs during normal development and homeostasis. **(A)** Early embryonic and fetal development are typically associated with high levels of YAP/TAZ activity, which are gradually downregulated after birth, as growth during the juvenile phase slows down, **(B)** finally reaching basal levels at the quiescent state of mature adult tissues/organs. **(C)** At the onset of tissue/organ injury, the mobilization and rapid proliferation of endogenous adult stem/progenitor cells are associated with highly elevated YAP/TAZ expression levels, which **(D)** return to basal levels of the quiescent state after organ/tissue healing and regeneration is completed.

It is important to note that under physiological conditions, the distribution of YAP/TAZ is usually partially cytoplasmic and partially nuclear, being dependent on the relative activities of the various components of the core Hippo signaling cascade (Figure 2) and other non-Hippo signaling pathways (Pocaterra et al., 2020). It is precisely this delicate balance of nuclear to cytoplasmic ratio of unphosphorylated and phosphorylated YAP/TAZ respectively, which plays such a crucial role in cell lineage fate determination (Figure 3); and in the activation and mobilization of endogenous stem/progenitor cells during the regeneration process following tissue/organ disease or injury (Figure 4). Nevertheless, it must be noted that there are additional layers of complex mechanisms that modulate the effects of YAP/TAZ signaling via (i) factors that modulate YAP/TAZ binding to TEAD, such as P38 MAPK4 (Lin et al., 2017a) and VGLL4 (Zhang et al., 2014; Lin et al., 2016; Deng and Fang, 2018); (ii) methylation and phosphorylation of YAP/TAZ via SET7 (Oudhoff et al., 2013) and PTPN14 (Liu et al., 2013) respectively; and (iii) YAP/TAZ binding and modulation of various non-TEAD transcription factors, such as Smad2/3 (Grannas et al., 2015), Runx2 (Brusgard et al., 2015; Lin et al., 2019), p63 (Tomlinson et al., 2010), p73 (Roperch et al., 2008), PRDM4 (Liu et al., 2018), OCT4 and SOX2 (Bora-Singhal et al., 2015).

As mentioned earlier, YAP/TAZ controls the expression of target genes primarily by acting as a co-activator of TEAD transcription factors, of which there are four isoforms in mammals (TEAD1-4) (Holden and Cunningham, 2018). The various target genes regulated by YAP/TAZ through TEAD1-4 regulate a wide range of key biological processes, which can be broadly classified into the following six categories (Moya and Halder, 2019): (i) cell proliferation, cell cycle and tumorigenesis, (ii) cell migration, (iii) stemness/dedifferentiation, (iv) cell lineage fate determination and differentiation, (v) cytoskeleton and cell morphology, and (vi) anti-apoptosis and cell survival (Moya and Halder, 2019). These are all of much relevance and interest to tissue engineering and regenerative medicine applications.

Upstream signaling pathways regulating YAP/TAZ activity have been shown to mediate cellular interactions with a broad range of microenvironmental factors including (i) soluble bioactive ligands (Yu et al., 2012; Cai and Xu, 2013; Chen and Harris, 2016; Yang et al., 2019), (ii) biomechanical cues (Dupont et al., 2011; Kim et al., 2011; Nardone et al., 2017; Pardo-Pastor et al., 2018), (iii) energy, osmotic and hypoxic stress (DeRan et al., 2014; Ma et al., 2015; Mo et al., 2015), and (iv) inflammation and tissue injury (Gregorieff et al., 2015; Kim H. B. et al., 2017; Choi et al., 2018; Flinn et al., 2019), via a diverse array of surface receptors, cytoskeletal elements and cytosolic signaling proteins, as illustrated in Figure 2. Hence, by manipulating YAP/TAZ signaling, we can control how stem/progenitor cells change their phenotype in response to external stimulation and microenvironmental cues. Of particular interests to the tissue engineering and regenerative medicine fields are how YAP/TAZ mediate cellular interactions with the native extracellular matrix and newly developed synthetic biomaterials, as well as adapt cells to pathological conditions at disease and injury sites.

Hence the focus of this review would be on the regulatory mechanisms by which YAP/TAZ regulate stem/progenitor cell differentiation into the major lineages that are of interest for therapeutic applications (Table 1). The key roles of YAP/TAZ in maintaining the delicate balance between quiescence, self-renewal, proliferation and differentiation of endogenous adult stem cells within various tissues/organs during early development, normal homeostasis and regeneration/healing will be critically examined (Figure 4). Finally, we will consider how increasing knowledge of YAP/TAZ signaling mechanisms might influence the trajectory of future progress in regenerative medicine, particularly therapeutic applications of stem cells.

**TABLE 1:** Major signaling pathways that interact with or modulate YAP/TAZ in various cell and tissue lineages, which in turn effect cell fate decisions such as proliferation, differentiation, and maintenance of “stemness.”

**Cell/tissue lineage**	**Major signaling pathways that interact with or modulate YAP/TAZ**	**Species/cell type**	**Key references**
Maintenance of pluripotency and “stemness”	Wnt/β-catenin	Murine ESCs	Papaspyropoulos et al. (2018)
	AKAP-Lbc/Rho-GTPase	Human ESCs	Ohgushi et al. (2015)
	FAK-YAP-mTOR	Human ESCs	Hu et al. (2017)
	Snail/Slug	Murine MSCs/murine skeletal stem cells	Tang and Weiss (2017)/Tang et al. (2016)
Bone/osteogenic lineage	Wnt/β-catenin	Murine MSCs	Seo et al. (2013); Pan et al. (2018)
	MIF/Akt (protein kinase B)	Human MSCs	Yuan et al. (2016)
	JNK	Human MSCs	Barreto et al. (2017)
	FAK/MAPK	Human MSCs	Hwang et al. (2015, 2017)
	Rho GTPase	Human MSCs	Qian et al. (2017)
	Snail/Slug	Murine MSCs/murine skeletal stem cells	Tang and Weiss (2017)/Tang et al. (2016)
Fat/Adipogenic lineage	PPARγ	Murine MSCs	Hong et al. (2005)
Cartilage/chondrogenic lineage	Wnt/β-catenin	Murine ATDC5 cells/Bovine chondrocytes	Yang et al. (2017)/Öztürk et al. (2017)
Nerves/Neural lineage	Sonic hedgehog	Human medulloblastomas and murine neural progenitors/murine P19 cells	Fernandez-L et al. (2009)/Lin et al. (2012)
	Wnt/β-catenin	Rat NSCs/human iPSCs	Rammensee et al. (2017)/Bejoy et al. (2018)
	Rho GTPase	Human SH-SY5Y cells	Baek et al. (2018)
Astrocytes	SMAD1/5/8 signaling	Murine NSCs and astrocytes	Huang et al. (2016)
Kidney/nephrogenic lineage	Rho GTPase	Murine nephron progenitors	Reginensi et al. (2013)
	FAT4	Murine nephron progenitors	Murphy et al. (2014)
Blood vessels/Angiogenesis and Endothelial lineage	MYC	HUVEC	Kim J. et al. (2017)
	BMP	HUVEC	Neto et al. (2018)
	PI3K-AKT	HUVEC	Choi and Kwon (2015)
	VEGF	Various human and murine endothelial cell types	Wang X. et al. (2017), (Elaimy and Mercurio, 2018)
	Rho GTPase	HUVEC	Sakabe et al. (2017)
	PGC1α	HUVEC	Mammoto et al. (2018)
	STAT3	HUVEC and breast tumor fibroblasts	Du et al. (2017)
Liver/hepatic lineage	TGF-β1	Murine hepatoblasts/hepatic stellae cells	Lee et al. (2016)/Yu et al. (2019)
	Wnt/β-catenin	Murine hepatic stellae cells	Yu et al. (2019)
	Notch	Murine hepatocytes	Tharehalli et al. (2018)
	Sonic Hedgehog	Murine hepatic stellae cells	Swiderska-Syn et al. (2016); Du et al. (2018)
Skeletal muscles/myogenic lineage	CREB-MPP7-AMOT	Murine muscle satellite cells	Li and Fan (2017)
	MEK5-ERK5	Murine C2C12 myoblasts	Chen et al. (2017)
Cardiac muscles/cardiomyogenic lineage	Insulin-like growth factor	Murine embryonic cardiomyocytes	Xin et al. (2013)
	Wnt/β-catenin	Murine embryonic cardiomyocytes	Xin et al. (2013)
	TAOK1	Rat neonatal cardiomyocytes	Torrini et al. (2019)
	E3 ubiquitin ligase β-TrCP	Rat neonatal cardiomyocytes	Torrini et al. (2019)
	PI3K-AKT	Rat neonatal cardiomyocytes	Lin et al. (2015)
	Wnt/β-catenin	Human ESCs	Estarás et al. (2017)
Skin/epidermal and keratinocyte lineages	Notch	Human epidermal keratinocytes	Totaro et al. (2017)
	α-Catenin	Murine embryonic keratinocytes	Schlegelmilch et al. (2011)
	Integrin-Src	Human keratinocytes	Elbediwy et al. (2016)
	Wnt/β-catenin	Human HaCaT keratinocyte cells/murine karatinocytes	Mendoza-Reinoso and Beverdam (2018)/Akladios et al. (2017b)
	GLI2/Hedgehog	Murine keratinocytes	Akladios et al. (2017a)
Intestinal epithelium/epithelial lineage	Rho GTPase	Murine intestinal epithelial cells	Liu et al. (2017)
	Epiregulin/EGFr	Murine intestinal epithelial cells	Liu et al. (2017)
	Wnt/β-catenin	Murine intestinal epithelial cells	Zhou et al. (2011); Barry et al. (2013), Llado et al. (2015)

## Role of Yap/Taz in Stem Cell Self-Renewal and Maintenance of Stem Cell Phenotype

Stem cells are defined by two key characteristics: (i) potency, which is the ability to give rise to multiple differentiated lineages, and (ii) capacity for self-renewal. Currently, four broad categories of stem cells are recognized: (i) adult stem cells, (ii) fetal stem cells, (iii) embryonic stem cells (ESCs), and (iv) induced pluripotent stem cells (iPSCs) derived by reprogramming somatic cells. Of these, ESCs and iPSCs are considered to be pluripotent and are collectively referred to as pluripotent stem cells. Adult and fetal stem cells, on the other hand, are considered to be only multipotent. It must be noted that adult stem cells are closely associated with cancer, and there is a widely accepted theory that cancer cells are in fact aberrant adult stem cells that have lost their capacity for proper regulation of apoptosis and the cell cycle (Zanconato et al., 2016). Indeed, both cancer cells (Chen et al., 2019) and stem cells (Lian et al., 2010) are known to exhibit increased YAP/TAZ activity.

Lian et al. (2010) reported that YAP activity is downregulated at the onset of mouse ESC differentiation, and that YAP gene silencing leads to a loss of ESC pluripotency. On the other hand, ectopic YAP expression blocks ESC differentiation *in vitro* and maintains the stem cell phenotype even under differentiation conditions. Subsequently, Tamm et al. (2011) showed that YAP and TEAD2, which are highly expressed in self-renewing mouse ESCs, are activated by both LIF (leukemia inhibitory factor) and serum, and that TEAD2 associates directly with the promoter of OCT3/4, a well-known pluripotency gene marker. Inter-α-inhibitor (IαI) was subsequently identified as the component in serum that can facilitate YAP activation and induce expression of the pluripotency markers OCT3/4 and Nanog in murine ESCs (Pijuan-Galitó et al., 2014). More recently, Papaspyropoulos et al. (2018) implicated YAP in the switch between pluripotency and differentiation in mouse ESCs. The tumor suppressor RASSF1A can block YAP from being an integral component of the β-catenin-TCF pluripotency network. At the onset of differentiation, demethylation of the Rassf1A promoter enables GATA1-mediated RASSF1A expression, which blocks YAP from contributing to the TEAD/β-catenin-TCF3 complex that maintains pluripotency in mouse ESCs (Papaspyropoulos et al., 2018).

It must be noted that the self-renewal and maintenance of pluripotency in human ESCs involve a different mechanism based on bFGF (basic fibroblast factor) signaling, as opposed to LIF signaling in mouse ESCs (Xu et al., 2005). Hence, findings with mouse ESCs may not necessarily be applicable to human ESCs. Nevertheless, several studies have demonstrated that human ESCs also exhibit elevated YAP/TAZ activity like mouse ESCs, which in turn plays a key role in their self-renewal, and maintenance of pluripotency and stem cell phenotype (Varelas et al., 2008; Musah et al., 2014; Ohgushi et al., 2015; Hsiao et al., 2016). Varelas et al. (2008) demonstrated that TAZ is required to maintain self-renewal of human ESCs and that downregulation of TAZ leads to differentiation into the neuroectoderm lineage. Musah et al. (2014) found that stiff hydrogel matrices promote activation of YAP/TAZ, which in turn enables maintenance of human ESC pluripotency. Ohgushi et al. (2015) demonstrated that the long-term survival, self-renewal and proliferation of human ESCs in *in vitro* culture depend on the maintenance of YAP/TAZ activity through AKAP-Lbc/Rho GTPase signaling, which modulates actin microfilament organization. Hsiao et al. (2016) shed light on why neuroepithelial differentiation of human ESCs is induced at high cell densities, showing that at higher cell densities, YAP phosphorylation and translocation to the cytosol are increased. As a result, YAP-mediated maintenance of pluripotency is impeded, and neuroepithelial differentiation is induced (Hsiao et al., 2016).

Perhaps the most compelling evidence of the role of YAP/TAZ in the self-renewal and pluripotency of human pluripotent stem cells comes from studies on reprogramming adult somatic cells into iPSCs. Zhao et al. (2017) reported that when YAP is ectopically expressed, only two reprogramming factors - Oct4 and Sox2 - instead of the usual four reprogramming factors (Oct4, Sox2, c-Myc and Klf4) are required to reprogram human amniotic epithelial cells into iPSCs. Qin et al. (2012) showed that knockdown of LATS2, a key component of the Hippo pathway involved in the phosphorylation of YAP, which facilitates its retention within the cytosol, could increase the efficiency of reprogramming of human somatic cells into iPSCs. A further study by the same group (Qin et al., 2016) showed that recombinant overexpression of YAP in human ESCs and iPSCs promotes generation of the naive pluripotent stem cell state, which corresponds to a pre-implantation stage of development that is difficult to capture and sustain *in vitro*.

Besides pluripotent stem cells, YAP/TAZ also play key roles in the expansion, self-renewal and maintenance of “stemness” of tissue-specific adult stem cells. Upon disease or injury, the normally quiescent adult stem cells resident within specific tissues are mobilized into a “transit-amplifying” stage, in which they undergo rapid and extensive proliferation as an undifferentiated intermediate, prior to terminal differentiation into functional lineages. Utilizing the continuously growing mouse incisor model, Hu et al. (2017) identified a FAK-YAP-mTOR signaling axis that regulates entry into the transit amplifying stage and inhibits differentiation. The role of YAP/TAZ in adult stem cell mobilization and expansion during tissue injury is further supported by the finding that siRNA knockout of YAP/TAZ in full-thickness skin wounds impedes healing and regeneration (Lee et al., 2014).

Tang et al. (2016) and Tang and Weiss (2017) demonstrated that binding interactions between YAP/TAZ and the zinc-finger transcription factors Snail/Slug play a crucial role in regulating the self-renewal and differentiation of bone-marrow derived MSCs. Panciera et al. (2016) showed that transient expression of exogenous YAP or TAZ reprograms primary differentiated mouse cells into a tissue-specific stem/progenitor cell state. Various differentiated lineages of mouse cells, such as mammary gland, neuronal, and pancreatic exocrine cells, were efficiently reprogrammed to proliferative cells with stem/progenitor-like properties upon recombinant YAP overexpression (Panciera et al., 2016). Other adult stem cells in which YAP plays an integral role in self-renewal and maintenance of the “stemness” phenotype include neural stem cells (Han et al., 2015; Bao et al., 2017), muscle satellite cells (Judson et al., 2012), and intestinal stem cells (Imajo et al., 2015; Kim H. B. et al., 2017). These will be discussed in greater detail in sections “Role of YAP/TAZ in Neurogenesis and Neuroregeneration,” “Role of YAP/TAZ in Myogenic Differentiation and Skeletal Muscle Regeneration,” and “Role of YAP/TAZ in Intestinal Epithelium Differentiation and Regeneration,’ respectively.

## Modulation of Stem/Progenitor Cell Lineage Fate by YAP/TAZ

### Role of YAP/TAZ in Osteogenesis and Bone Regeneration

There is abundant evidence for key roles of YAP/TAZ in the osteogenic differentiation pathway of primary osteoblasts and various types of adult stem cells, especially bone marrow-derived MSCs. While TAZ activation is consistently associated with osteogenesis (Hong et al., 2005; Suh et al., 2012, 2014; Yang et al., 2013; Zhu Y. et al., 2018), the role of YAP in osteogenic differentiation is controversial (Seo et al., 2013; Sen et al., 2015; Pan et al., 2018; Zhu W. Q. et al., 2018; Bai et al., 2019; Lin et al., 2019; Wei et al., 2019).

The role of TAZ in the osteogenesis of MSCs was first reported by Hong et al. (2005), who found that TAZ co-activates gene transcription by RUNX2, an upstream regulator of osteogenesis, while at the same time repressing gene transcription by PPARγ that directs murine bone marrow MSCs into the adipocyte lineage. Subsequent studies provided unambiguous evidence that TAZ activation promotes osteogenic differentiation. Yang et al. (2013) found that whole-body bone mineral density (BMD) is significantly increased in transgenic mice that overexpress TAZ, as compared to wild-type mice. Suh et al. (2012, 2014) genetically engineered a recombinant cell-permeable TAZ fusion protein that promoted osteogenesis of human dental pulp stem cells (Suh et al., 2012) and MSCs (Suh et al., 2014), while at the same time inhibiting adipogenic differentiation of these cells. In accordance with the results of those studies, TAZ knockdown in mice impairs osteogenic differentiation, but enhances adipogenic differentiation of human adipose-derived stem cells (Zhu Y. et al., 2018).

The role of YAP in osteogenesis is ambiguous. While some studies indicated that YAP activation enhances osteogenesis, other studies reported contrary results. In two separate studies by our research group, we found that YAP activation in rat bone marrow-derived MSCs is associated with osteogenesis (Bai et al., 2019; Wei et al., 2019). Pan et al. (2018) demonstrated that conditional knockout of YAP expression by the osteoblast lineage in mice reduced osteogenic differentiation and cell proliferation, but promoted adipocyte formation, which in turn led to trabecular bone loss. Furthermore, recombinant β-catenin expression in YAP-deficient bone marrow stromal cells diminished this osteogenesis deficit, which may imply that YAP promotes osteogenesis via the Wnt/β-catenin signaling pathway. However, contrary data were obtained in other studies. Seo et al. (2013) reported that YAP binding to β-catenin directly induces the Wnt antagonist Dkk1 to dampen pro-osteogenic Wnt signals in murine MSCs. Similarly, Sen et al. (2015) found that maintenance of YAP nuclear translocation inhibits Runx2 initiation of osteogenesis in human and murine bone marrow MSCs, and Zhu W. Q. et al. (2018) reported that titanium ion-induced YAP activation downregulates osteogenic differentiation of murine MC3T3-E1 cells.

The findings of Lin et al. (2019) may shed some light on the ambiguous role of YAP in regulating osteogenesis. They found that YAP binds to and inhibits the upstream pro-osteogenic transcription factor RUNX2 in human bone marrow and dental-derived MSCs, and that this inhibition is released by competitive binding of AP2a (activator protein 2a) to YAP (Lin et al., 2019). Thus, YAP binding and inhibition of RUNX2 may serve as a negative feedback mechanism to YAP activation of RUNX transcription through TEAD, and AP2a may provide a release from this negative feedback mechanism. Seo et al. (2013) reported that a delicate balance of YAP and SOX2 in murine bone marrow MSCs regulates differentiation into either the adipogenic or osteogenic lineages, and that osteogenesis is inhibited by high SOX2 or YAP1, but enhanced by depletion of either one. The conflicting results on the role of YAP in osteogenesis may be due in part to differences in the cell lineages and species examined in the various studies. Also, there may be other, as-yet-undiscovered, negative feedback mechanisms that control the YAP modulation of osteogenic differentiation, as well as other endogenous signaling molecules that modulate YAP function, such as AP2a (Lin et al., 2019), in the case of human bone marrow and dental-derived MSCs. Further studies are needed to unravel the role of YAP in osteogenesis.

YAP/TAZ regulation of osteogenic differentiation is stimulated by various physical and biochemical stimuli. The most widely studied of these are substrate stiffness and surface topography. Generally, stiff and rigid substrates that are conducive to cell adhesion and spreading lead to increased formation of FAs and cytoskeletal stress fibers, which in turn facilitate nuclear translocation of YAP/TAZ through both Hippo-dependent and Hippo-independent mechanisms. This in turn promotes osteogenic differentiation whilst inhibiting adipogenesis, through simultaneous activation and inhibition of RUNX2 and PPARγ (peroxisome proliferator-activated receptor gamma) transcription, respectively (Pan et al., 2017; Oliver-De La Cruz et al., 2019). On the other hand, soft substrates that are not conducive to cell adhesion and spreading lead to decreased formation of FAs and cytoskeletal stress fibers. These changes decrease YAP/TAZ activity and consequently inhibit osteogenesis while promoting adipogenic differentiation (Pan et al., 2017; Oliver-De La Cruz et al., 2019).

There is also evidence that a multitude of other signaling pathways are interwoven in substrate-stiffness-induced osteogenesis. Yuan et al. (2016) showed that YAP/TAZ activation and promotion of osteogenic differentiation on a stiff substrate may be mediated by macrophage migration inhibitory factor (MIF) and the Akt (protein kinase B) signaling pathway, while Barreto et al. (2017) implicated the JNK signaling pathway in stiffness-induced osteogenesis of MSCs. Hwang et al. (2015) showed that extracellular matrix stiffness regulates osteogenic differentiation of MSCs via TAZ activation through the MAPK signaling pathway. In an interesting study by Yang et al. (2014), it was demonstrated that previous culture on a stiff substrate could bias human MSCs toward YAP/TAZ activation and osteogenic differentiation upon subsequent culture on soft substrates, which may mean that cells possess a form of “mechanical memory.”

With regard to substrate surface topography, several studies have demonstrated conclusively that rough, patterned and fibrous substrates are more conducive to YAP/TAZ activation, which in turn enhances osteogenic differentiation. Yang W. et al. (2016) demonstrated that YAP/TAZ activation and osteogenic differentiation of bone marrow-derived MSCs were optimal on hydroxyapatite discs with a surface roughness of 0.77 to 1.09 μm, and with a mean distance between peaks of 53.9 to 39.3 μm. Both micropatterned and nanopatterned substrates also enhance osteogenesis via YAP/TAZ activation. Zhang Y. et al. (2016) showed that YAP activation and osteogenic differentiation of MC3T3-E1 cells were enhanced on polydimethylsiloxane (PDMS) micropatterns with grid topology, while Hwang et al. (2017) and Qian et al. (2017) showed that substrate nanotopographical features can enhance osteogenesis of MSCs through increased TAZ activation. The underlying mechanisms of surface topology-induced osteogenesis by patterned and fibrous substrates are thought to be correlated to surface roughness. Generally, evidence from several studies (Yang W. et al., 2016; Zhang Y. et al., 2016; Hwang et al., 2017; Qian et al., 2017) suggest that a rougher surface (i.e., deeper grooves, higher peaks and troughs, greater spacing between topographical features and random alignment of fibers) promotes stronger adhesion and greater cell spreading through increased integrin clustering and FA formation. This in turn enhances actin polymerization and increases cytoskeletal tension via Rho GTPase signaling, as well as by the FAK and MAPK signaling pathways, ultimately increasing YAP/TAZ nuclear translocation that promotes osteogenesis (Yang W. et al., 2016; Zhang Y. et al., 2016; Hwang et al., 2017; Qian et al., 2017). Hence, it can be concluded that osteogenesis will be enhanced on any substrate in which cells display stronger adhesion and greater spreading via increased integrin clustering and FA formation. For example, Arslan et al. (2017) demonstrated conclusively that nanofiber morphology exerts a profound effect on cellular YAP activation and osteogenesis via modulation of integrin clustering. In particular, cylindrical peptide nanofibers facilitated the formation of integrin β1-based FA complexes, which in turn enhanced the osteogenic potential of stem cells through increased YAP activation, whereas twisted ribbon-like nanofibers had the opposite effect (Arslan et al., 2017). Likewise, in an interesting study by Wang et al. (2016), micropatterned substrates enabled precise control of the spreading and adhesion area of human MSCs, and it was found that a larger cell adhesion area promoted both YAP/TAZ activation and osteogenesis, while a smaller cell adhesion area reduced TAP/TAZ activity and promoted adipogenic differentiation. In contrast, the total cell spreading area *per se* (not adhesion area) did not have any effect on YAP/TAZ activity or cell differentiation.

Besides substrate stiffness and surface topography, various biophysical stimuli also modulate osteogenesis via YAP/TAZ. These include mechanical stimuli, such as cyclic stretching (Yang et al., 2018), shear stress (Kim et al., 2014), acoustic tweezing (Xue et al., 2017), pH (Tao et al., 2016), microgravity (Chen et al., 2016) and light (Feng et al., 2015). Various exogenous growth factors and protein ligands, such as IRS-1 (insulin receptor substrate 1) (Wang N. et al., 2018), FGF2 (fibroblast growth factor-2) (Byun et al., 2014a), CTHRC1 (collagen triple helix repeat containing 1) (Wang C. et al., 2017), FBLN1 (fibulin-1) (Hang Pham et al., 2017), IGF1 (insulin-like growth factor 1) (Xue et al., 2013), and TGF-β1 (transforming growth factor beta one) promote osteogenesis via TAZ activation (Zhao et al., 2010). However, TNF-α (tumor necrosis factor-alpha) was reported to suppress TAZ activation and to impair the osteogenic potential of MSCs (Li et al., 2007). Similarly, various pharmacologically active chemicals promote osteogenesis through activation of TAZ. These include TM-25659 (Jang et al., 2012), sodium butyrate (Fan et al., 2018), lipopolysaccharide (Xing et al., 2019), 1α,25-dihydroxyvitamin D3 (Ji et al., 2019), icariin (Wei et al., 2017; Ye et al., 2017), epicatechin gallate (ECG) (Byun et al., 2014b), phorbaketal A (Byun et al., 2012a), kaempferol (Byun et al., 2012b), and poncirin (Yoon et al., 2011).

The regulation of osteogenesis by YAP/TAZ involves various intracellular signaling proteins that interact directly with YAP/TAZ. For example, Smad4 (Park et al., 2019) and polycystin-1 bind directly with TAZ and facilitate its nuclear translocation (Xiao et al., 2018). As described earlier, an intracellular protein that binds directly with YAP is AP2a, with formation of the YAP-AP2a protein complex releasing YAP binding and inhibition of RUNX2 activity (Lin et al., 2019). Additionally, the YAP-AP2a protein complex blocks transcription of BARX1, which inhibits osteogenesis (Lin et al., 2019). Snail and Slug are zinc-finger transcription factors that interact directly with both YAP and TAZ, thereby promoting osteogenesis via activation of RUNX2 transcription (Panciera et al., 2016; Tang et al., 2016). Another mechanism through which intracellular signaling proteins modulate osteogenesis via YAP/TAZ is by regulating the assembly of FAs and stress fibers, which is interlinked with RhoA activity. As mentioned earlier, RhoA is a GTPase in the Rho family that is involved in actin polymerization and formation of FAs (Buhl et al., 1995; Seo et al., 2011). RhoA is involved in YAP/TAZ activation by modulating the phosphorylation of LATS1/2, which is a key component of the Hippo signaling pathway (Ohgushi et al., 2015). Guo et al. (2018) reported that kindlin-2 binds with myosin light-chain kinase in response to mechanical cues, leading to myosin light-chain phosphorylation. This facilitates assembly of FAs and stress fibers, as well as activation of RhoA, which in turn promotes osteogenic differentiation via YAP/TAZ activation. Studies involving recombinant overexpression, gene knockout and inhibition have identified a number of other intracellular signaling proteins that modulate osteogenesis via regulation of YAP/TAZ activity. These includes RAMP1 (receptor activity-modifying protein 1) (Hwang et al., 2017), αCGRP (α-calcitonin gene-related peptide) (Xiang et al., 2019), TWIST1 (Quarto et al., 2015), PP1A (protein phosphatase 1A) and NF-kappaB (Cho et al., 2010). Recently, Li C. J. et al. (2018) identified a long non-coding RNA, Bmncr, that facilitates the assembly of the TAZ and RUNX2/PPARG transcriptional complex, which promotes osteogenesis of MSCs.

### Role of YAP/TAZ in Adipogenesis

As discussed in the previous section, YAP/TAZ nuclear translocation plays a key role in determining whether MSCs differentiate into either the osteogenic or adipogenic lineage. As in the case of osteogenic differentiation, there is some controversy concerning the role of YAP in adipogenesis. Chang et al. (2017) reported that recombinant overexpression of YAP in 3T3-L1 pre-adipocytes suppresses adipogenesis. Similarly, Pan et al. (2018) reported that YAP knockdown in mouse osteoblasts promotes adipogenic differentiation. However, Kamura et al. (2018) reported increased obesity in YAP-overexpressing transgenic mice, which appears to imply that YAP activation promotes adipogenesis. Nevertheless, further investigations on the adipose stem cells of these transgenic mice *in vitro* revealed that YAP overexpression induces a negative feedback mechanism on the Hippo signaling pathway, which leads to suppression of TAZ activity. This in turn enhances PPARγ activation and increases adipogenesis (Kamura et al., 2018). Hence, this negative feedback mechanism might account for the apparently conflicting findings regarding the role of YAP in regulating differentiation to the adipogenic and osteogenic lineages. In contrast, the role of TAZ in adipogenesis seems clear. TAZ activation inhibits adipogenesis through suppression of PPARγ transcription (Hong et al., 2005; Kawano et al., 2015), whereas TAZ inactivation promotes adipogenic differentiation through increased PPARγ transcription (Hong et al., 2005; Kawano et al., 2015).

As discussed in the previous section, mechanosensing of substrate stiffness via YAP/TAZ regulates differentiation of MSCs into either the adipogenic or osteogenic lineage. But, contrary to what occurs in the case of osteogenesis, adipogenic differentiation is promoted on soft substrates that are non-conducive to cell adhesion and formation of FAs and stress fibers (Ji et al., 2019; Xing et al., 2019). On stiff substrates, the cytoskeletal focal adhesion protein vinculin promotes nuclear localization of TAZ, which inhibits adipogenic differentiation (Kuroda et al., 2017). On the other hand, Loye et al. (2018) demonstrated that reduced cell adhesion on nanopatterned bulk metallic glass, which promotes a more rounded morphology, reduces YAP activity and promotes the adipogenic differentiation of MSCs. Similarly, inhibition of cellular adhesion to nascent proteins deposited on hydrogels favors adipogenesis by reducing YAP/TAZ activity (Loebel et al., 2019). In addition, Morandi et al. (2016) observed that adipogenic differentiation is associated with downregulation of RGD-motif binding integrin-alpha-V (ITGAV) and integrin-alpha-5 (ITGA5), both of which are key components of FAs.

Exogenous growth factors and proteins that promote adipogenesis through suppression of YAP/TAZ activity include liraglutide (Li Y. et al., 2018) and sclerostin (Ukita et al., 2016), while small molecules that exert similar pharmacological effects include thiazolidinedione (Basu-Roy et al., 2016) and dexamethasone (He et al., 2012).

### Role of YAP/TAZ in Chondrogenesis and Cartilage Regeneration

The overwhelming majority of reported studies indicate that YAP/TAZ activation is associated with inhibition of chondrogenic differentiation and promotion of chondrocyte proliferation, while reduced YAP/TAZ activity is associated with induction of chondrogenesis and suppression of chondrocyte proliferation. Karystinou et al. (2015) observed that in developing mouse limbs, YAP localization is mainly nuclear in the perichondrium, while YAP is mostly phosphorylated and localized within the cytosol of cells in the cartilage anlage. This would suggest that there is decreased YAP activity during physiological chondrogenesis *in vivo*. Further, Karystinou et al. (2015) also demonstrated that YAP, but not TAZ, is deactivated during *in vitro* chondrogenesis of human MSCs, and that recombinant overexpression of human YAP in murine C3H10T1/2 MSCs inhibits chondrogenesis. Similarly, Goto et al. (2018) demonstrated that hyperactivation of endogenous YAP/TAZ impairs chondrocyte differentiation and maturation, leading to chondrodysplasia in Mob1a/b-deficient mice; and that this is linked to suppression of SOX9, an upstream regulator of chondrogenesis. Deng et al. (2016) showed that YAP promotes early chondrocyte proliferation through direct upregulation of Sox6 expression, but inhibits subsequent chondrocyte maturation both *in vitro* and *in vivo* by suppressing Col10a1 expression through interaction with Runx2. Utilizing the mouse chondroprogenitor ATDC5 cell line, Yang et al. (2017) also demonstrated that YAP overexpression promotes chondrocyte proliferation, but inhibits chondrocyte differentiation through the Wnt/β-catenin signaling pathway. The role of Wnt/β-catenin signaling in suppressing chondrogenic differentiation was confirmed by Öztürk et al. (2017), who showed that β-catenin is upregulated in de-differentiating chondrocytes. Öztürk et al. (2017) also observed that chondrocyte de-differentiation is accompanied by increased RhoA activity.

As in the case of osteogenesis and adipogenesis, chondrogenic differentiation is also sensitive to biomechanical cues such as substrate stiffness and surface topology. Zhong et al. (2013a) found that the differentiated chondrocyte phenotype was maintained on soft substrates through reduction of YAP activity, which led to inhibition of chondrocyte proliferation. By contrast, YAP activation on stiff substrates promotes chondrocyte de-differentiation and proliferation (Zhong et al., 2013a). On fibrous substrates, the chirality and morphology of the nanofibers have a profound effect on chondrogenic differentiation. Arslan et al. (2017) found that the d-form of twisted-ribbon like nanofibers (d-FF) enhanced the chondrogenic potential of stem cells more than their l-form (l-FF) by guiding the cells into round shapes and decreasing the formation of FA complexes (Arslan et al., 2017). Besides the mechanical properties of the substrata, chondrocytes are also sensitive to mechanical forces. Zhong et al. (2013b) reported that MSCs and chondrocytes subjected to shear forces within a microfluidic perfusion device exhibited increased YAP activation, which in turn led to chondrocyte de-differentiation and promoted osteogenesis in MSCs. Yang K. et al. (2016) reported that when chondrocytes derived from the growth plate cartilage of 2-week-old rats were exposed to mechanical stress, YAP activation was increased, and this facilitated cell cycle progression through RhoA and cytoskeletal dynamics.

Nevertheless, there are some contradictory results indicating that YAP activation is associated with promotion of chondrogenic differentiation. These discrepancies may have arisen from different developmental stages of the studied stem cells, i.e., embryonic versus adult stem cells, or from differences in the specific types of stimuli used to promote chondrogenic differentiation through YAP activation. For example, in the case of embryonic stem cells, which are at an earlier and less mature developmental stage than adult stem cells, compressive mechanical stress upregulate expression of chondrogenic markers such as collagen type 2, Sox9 and aggrecan, concomitantly with increased YAP/TAZ and RhoA activity (McKee et al., 2017). A specific stimulus that has been reported to maintain differentiated chondrocyte phenotype via YAP activation is hypoxia (Li H. et al., 2018). HIF-1α (hypoxia-inducing factor 1 alpha) is involved in this process, because inhibition of HIF-1α expression decreases YAP activation and downregulates SOX9 expression under hypoxic conditions (Li H. et al., 2018). On the other hand, upregulation of HIF-1α by cobalt chloride enhances YAP activation and increases the expression of collagen II and SOX9 under normoxic conditions (Li H. et al., 2018). Another specific stimulus that promotes chondrogenic differentiation through YAP activation is shear force perpendicular to aligned nanofibers on which MSCs are cultured (Zhong et al., 2013c), with RhoA being implicated in this process.

Similarly, there are conflicting data on the role of YAP in the progression of osteoarthritis. Deng et al. (2018) showed in a mouse model of experimental osteoarthritis that articular cartilage integrity can be preserved through YAP activation via transgenic overexpression or via deletion of it’s upstream inhibitory kinase MST1/2, whereas knockdown of YAP in chondrocytes promoted cartilage degradation. In contrast, Gong et al. (2019) found that suppression of YAP activity with siRNA prevents cartilage degradation and ameliorates osteoarthritis development in a mouse model. This discrepancy may have arisen because of the complex nature of osteoarthritis pathology, which remains to be fully understood.

### Role of YAP/TAZ in Neurogenesis and Neuroregeneration

Neural development in mammals involves initial formation of the embryonic neural crest, which not only gives rise to the neural lineage, but also various other cranio-facial lineages (Prasad et al., 2019). This is followed by lineage commitment to neural stem/progenitor cells, and subsequent further lineage specification into neurons, astrocytes, Schwann cells and oligodendrocytes (Prasad et al., 2019). Astrocytes, Schwann cells and oligodendrocytes, commonly referred to as glial cells, form the myelin sheath, maintain homeostasis of neural tissues, and provide support and protection for neurons (Prasad et al., 2019). YAP/TAZ play crucial roles at all these stages of neurogenesis during the development of the mammalian central and peripheral nervous systems.

Hindley et al. (2016) investigated the role of Hippo/YAP signaling in several neural cell lines, such as SH-SY5Y, LUHMES, NTERA2 and pluripotent stem cell-derived neural stem cells (NSCs), and found that YAP activity promotes an early neural crest phenotype, as well as migratory activities associated with the neural crest. Zhang et al. (2018) reported that YAP plays a crucial role in the induction of human gingiva-derived mesenchymal stem cells (GMSCs) into neural crest stem-like cells (NCSCs), as YAP knockdown attenuated the expression of NCSC-related genes. Maintenance of embryonic neural stem cell characteristics was shown to be dependent on YAP/TAZ activity mediated by TEAD (Han et al., 2015). Han et al. (2015) found that recombinant overexpression of YAP/TAZ increases the formation and size of neurospheres, which implies enhanced self-renewal and proliferative capacity of NSCs. These effects appear to be TEAD-dependent, because the capacity to induce neural stem cell characteristics was lost in a TEAD binding-defective YAP mutant. Similarly, Saito et al. (2018) observed that elevated expression of YAP or TEAD enhances the self-renewal and stem-like characteristics of neural progenitor cells.

It should be noted that brain development and homeostasis require a delicate balance between the expansion of neural stem/progenitor cells and differentiation into post-mitotic neurons and glia. Several studies have demonstrated that Hippo-YAP signaling plays a crucial role in this balancing act. Li et al. (2012) observed that YAP expression is limited to the stem cell compartment in the developing forebrain and that YAP expression rescues Notch pathway inhibition in NSC self-renewal assays (Li et al., 2012). Lavado et al. (2018) found that the Hippo pathway controls the number of neural progenitors within the developing mouse brain by blocking YAP/TAZ-driven hypertranscription. They also found that the tumor suppressor NF2 (merlin) restricts the expansion of neural progenitor cells (NPCs) by inhibiting YAP/TAZ activity through a Hippo-independent mechanism (Lavado et al., 2013). Similarly, Van Hateren et al. (2011), showed that FatJ cadherin acts via YAP inactivation through the canonical Hippo signaling pathway to limit the size of neural progenitor cell pools within the developing neural tube. Conversely, Cao et al. (2008) reported that increased YAP and TEAD activity leads to marked expansion of the neural progenitor population by facilitating cell cycle progression through induction of cyclin D1, as well as by inhibiting differentiation through suppression of NeuroM. YAP-mediated neural progenitor proliferation also involves Sonic Hedgehog (SHH) signaling (Fernandez-L et al., 2009). An interesting study by Ji et al. (2017) revealed that mitochondrial uncoupling protein 2 (UCP2) regulates the proliferation of neural progenitors by modulating the production of reactive oxygen species (ROS), which in turn controls YAP degradation through the ubiquitin-proteasome proteolytic pathway.

As in the cases of osteogenesis and adipogenesis, the modulation of neuronal differentiation by substrate stiffness and surface topography is also mediated via YAP/TAZ. As with adipogenesis, neuronal differentiation is favored on softer substrata with a low Young’s modulus, where nuclear translocation of YAP/TAZ is impeded (Thompson and Chan, 2016). Musah et al. (2014) showed that even in the presence of soluble pluripotency factors, compliant substrata promote highly efficient differentiation of human pluripotent stem cells into post-mitotic neurons by inhibiting YAP nuclear localization. Even without neurogenic factors, compliant substrata can produce neurons more rapidly and efficiently than conventional differentiation methods (Musah et al., 2014). By utilizing an oligonucleotide-crosslinked ECM platform that allows dynamic and reversible control of stiffness, Rammensee et al. (2017) demonstrated that YAP overexpression through substrate stiffening inhibits neural differentiation of NSCs, while suppressing YAP activity through substrate softening promotes neural differentiation. They also showed that ablating YAP-β-catenin interaction rescues neurogenesis on a stiff substrate, which may imply that ECM stiffness controls NSC lineage commitment by signaling via YAP and β-catenin interaction. Similarly, Sun et al. (2014) enhanced the purity and yield of functional motor neurons from human pluripotent stem cells after 23 days of culture by using soft microengineered substrate systems consisting of poly(dimethylsiloxane) micropost arrays (PMAs), while Catanesi et al. (2018) observed enhanced neurogenic differentiation on reduced graphene oxide (GO) materials that suppress YAP activation.

Baek et al. (2018) reported that Rho GTPase signaling is implicated in the mechanotransduction of substrate topography to lineage fate decisions in NSCs (Baek et al., 2018). Enhanced differentiation of NSCs was observed on a high-resolution nanogrooved substrate topography with an extremely narrow contact width that suppresses integrin clustering and FAs formation, and this in turn inhibits nuclear translocation of YAP via reduced Rho GTPase activity. Similarly, Song et al. (2016) found that neural differentiation of human iPSCs is enhanced on nanopatterned substrata with hexagonally arranged nanopillars (diameter of 560 nm) that suppress YAP activation, presumably through reduced formation of FAs. Besides substrata stiffness and surface topography, cell density is another biophysical cue that has been reported to modulate neuronal differentiation via YAP/TAZ. Hsiao et al. (2016) showed that at higher densities of human pluripotent stem cells, YAP phosphorylation and translocation to the cytosol increase, which in turn decrease YAP-mediated transcriptional activity. This promotes neuronal differentiation.

Some cell-surface receptor molecules have been reported to modulate neurogenic differentiation via YAP/TAZ. By means of gene silencing studies on SH-SY5Y cells, Ahmed et al. (2015) showed that FAT1 cadherin, which mediates intercellular contact via formation of AJs between adjacent cells, also plays a role in controlling neurite outgrowth, and drives SH-SY5Y cells toward terminal neural differentiation by inhibiting proliferation via TAZ deactivation. Metabotropic glutamate receptor 7 (GRM7) has been linked to brain developmental defects, such as attention deficit hyperactivity disorder (ADHD) (Septer et al., 2012). Knockout studies by Xia et al. (2015) demonstrated that GRM7 regulates neuronal differentiation by modulating YAP expression. Cytosolic signaling molecules have also been implicated in mediating the cross-talk of other signaling pathways with YAP/TAZ regulation of neurogenesis. Bejoy et al. (2018) observed Wnt and YAP interactions during neural tissue patterning of human induced pluripotent stem cells. Lin et al. (2012) demonstrated that YAP overexpression inhibits neuronal differentiation via the Sonic Hedgehog signaling pathway.

Hence, it can be concluded that, in general, increased YAP/TAZ activity is required for proliferation of neural stem/progenitor cells during the initial stages of neurogenesis, while reduced YAP/TAZ activity is required for differentiation into mature functional neurons during the later phase of neurogenesis. The stimulatory effect of YAP/TAZ activity on the proliferation of neural stem/progenitor cells may offer a basis for potential therapeutic strategies to promote neuroregeneration. For example, Bai et al. (2019) showed that inhibition of LATS1, a core component of the Hippo signaling pathway, increases YAP nuclear translocation, which in turn attenuates neuronal apoptosis and neurological impairment in a rat traumatic brain injury model. Also, Zhang et al. (2017) showed that stem cell niche-derived laminin-511 promotes midbrain dopaminergic neuron survival in response to oxidative stress through YAP activation, and that LM511-YAP signaling increases the expression of transcription factors associated with midbrain dopaminergic neuron identity, such as PITX3 and LMX1A.

Differentiation into functional glial cells is also mediated by YAP/TAZ. Huang et al. (2016) demonstrated that YAP is required for astrocytic differentiation of both NSCs and astrocytes, and that nuclear translocation of YAP is crucial for the stabilization of SMAD1/5/8 signaling during BMP2-induced astrocytic differentiation. Another study by Huang and Xiong (2016) indicated that the surface receptor protein neogenin is required for YAP-mediated astrocytic differentiation of NSCs and astrocytes, and is induced by BMP2. During Schwann cell development and myelination, YAP/TAZ regulate the expression of peripheral myelin protein 22 through TEAD1 (Lopez-Anido et al., 2016). Similarly, Poitelon et al. (2016) reported that YAP/TAZ regulate peripheral myelination and the expression of laminin receptors in Schwann cells. Grove et al. (2017) showed that nuclear translocation of YAP/TAZ is also necessary for developing Schwann cells to enter the S-phase and proliferate, and that YAP/TAZ regulate adult myelination by driving TEAD1 to activate the Krox20 transcription factor.

### Role of YAP/TAZ in Nephrogenesis and Kidney Regeneration

Nephrons, the functional filtration units within the kidney, arise from mesenchymal progenitors, and YAP/TAZ and the Hippo signaling pathway control the delicate balance between self-renewal and differentiation of mesenchymal progenitors that give rise to functional nephrons. Tanigawa et al. (2015) demonstrated that the SIX2+ nephron progenitor pool of the metanephric mesenchyme requires nuclear localization of YAP for proliferation and maintenance of nephron progenitor phenotype within *in vitro* culture. Mechanistically, the combination of LIF and Rho kinase inhibitor (ROCKi) supplemented in the culture milieu upregulates transcription factor SLUG expression, which in turn activates YAP, thereby maintaining SIX2, PAX2, and SALL1 expression by nephron progenitors of the metanephric mesenchyme. In accordance with the putative key role of YAP in the proliferation and phenotype maintenance of nephron progenitors, Reginensi et al. (2013) reported that YAP activation through mechanical stress transduced via Rho GTPase Cdc42 plays a crucial role in normal nephrogenesis during early mouse embryonic development. Murphy et al. (2014) found that knockdown of FAT4 signaling results in increased proliferation of nephron progenitors during embryonic kidney development through increased YAP nuclear localization and activation. This strongly resembles the dysregulation observed in Wilms tumor (WT), a type of embryonal malignancy with histological features reminiscent of the embryonic kidney.

Nevertheless, the subsequent deactivation of YAP/TAZ through phosphorylation is required for differentiation into mature functional nephrons. McNeill and Reginensi (2017) reported that LATS1/LATS2 knockdown in nephron progenitors of mice, which results in constitutive YAP activation, leads to disruption of nephrogenesis, as evidenced by an accumulation of spindle-shaped myofibroblastic cells in both the cortical and medullary regions of the kidney. They further showed that downregulation of YAP/TAZ expression levels can completely rescue the normal phenotype, and they concluded that YAP/TAZ deactivation through phosphorylation is required for further maturation of the nephron progenitors into functional nephrons. Similarly, Xu et al. (2016) found that kidney regeneration following acute injury (ischemia-reperfusion) is associated with dynamic regulation of YAP expression, and that YAP activation can have both beneficial and detrimental effects on kidney regeneration. On the one hand, YAP activation promotes repair of the injured kidney epithelia. On the other hand, excessive YAP activation might give rise to interstitial fibrosis and abnormal renal tubule differentiation. Anorga et al. (2018) also demonstrated that aberrant sustained TAZ activation confers a fibrotic maladaptive phenotype during kidney repair following injury.

Hence, it can be concluded that YAP/TAZ activation is required for proliferative expansion and phenotype maintenance of the nephron progenitor pool during both embryonic development and kidney regeneration, and that YAP/TAZ deactivation through phosphorylation is required for subsequent maturation of these nephron progenitors into functional nephrons.

### Role of YAP/TAZ in Angiogenesis and Vascularization

The formation of new blood vessels during the process of angiogenesis or vascularization is an extremely complex multi-step process that involves the coordinated migration and proliferation of endothelial cells (ECs) and smooth muscle cells (SMCs), followed by complex interactions among these cells and junction formation. Loss-of-function studies have confirmed that YAP/TAZ nuclear localization and activation are essential for initiating angiogenesis or vascularization. Singh et al. (2016) examined the early development of coronary vasculature, and found that YAP/TAZ inhibition disrupts epicardial epithelial-to-mesenchymal transition (EMT) and inhibits epicardial cell proliferation and differentiation into coronary ECs, in part through dysregulation of Tbx18 and Wt1 expression. Similarly, Kim J. et al. (2017) reported that endothelial-specific knockdown of YAP/TAZ leads to blunted-end, aneurysm-like tip ECs, with fewer and dysmorphic filopodia at the vascular front. Vascular network formation is inhibited, with reduced and disordered distributions of TJ and AJ proteins that disrupt barrier integrity. This in turn leads to hemorrhage in the growing retina and brain vessels, with reduced pathological choroidal neovascularization. In addition, Kim J. et al. (2017) showed that YAP/TAZ have multifaceted roles in angiogenesis. For example, YAP/TAZ coordinate EC proliferation and metabolic activity by upregulating MYC signaling, while at the same time regulating actin cytoskeleton remodeling during filopodia formation and junction assembly in ECs (Kim J. et al., 2017).

Neto et al. (2018) showed that YAP/TAZ regulate AJ dynamics and EC distribution during vascular development, in part by downregulating BMP signaling. Mechanistically, this involved a YAP/TAZ-mediated increase in the turnover of VE-cadherin, facilitating the formation of junction-associated intermediate lamellipodia, which promotes both cell migration and maintenance of barrier function. Subsequent knockdown of YAP/TAZ led to stunted sprouting, branching irregularities and junction defects. On the other hand, forced nuclear translocation of TAZ instead drives hypersprouting and vascular hyperplasia (Neto et al., 2018). Choi and Kwon (2015) showed that YAP activity is regulated by VE-cadherin-mediated contacts between ECs, modulated by the phosphoinositide 3-kinase-Akt signaling pathway. Furthermore, Choi and Kwon (2015) identified angiopoietin-2 (ANG-2) as a transcriptional target of YAP in regulating the angiogenic sprouting activity of ECs both *in vitro* and *in vivo*. Mammoto et al. (2019) reported that YAP also regulates the expression of the angiopoietin receptor Tie2.

Besides promoting the differentiation and angiogenic sprouting of ECs, YAP/TAZ modulate vascular smooth muscle cell (VMSC) proliferation and differentiation (von Gise et al., 2012; Feng et al., 2019). Wen et al. (2019) showed that YAP promotes VMSC differentiation by upregulating expression of the transcription factors Pitx2c and myocardin, while Osman et al. (2019) demonstrated that YAP promotes VMSC proliferation by upregulating SLC1A5 (solute carrier family 1 member 5)-mediated glutamine uptake.

YAP/TAZ interact with various canonical signaling pathways during angiogenesis and vascularization. The most prominent of these is the VEGF signaling pathway, which has a critical role in angiogenesis/vascularization. Several studies have reported the convergence of YAP/TAZ and VEGF signaling via the actin cytoskeleton (Wang X. et al., 2017; Elaimy and Mercurio, 2018). For example, VEGF stimulates Rho-GTPase activity, thereby altering cytoskeletal dynamics, which contributes to YAP/TAZ activation (Wang X. et al., 2017; Elaimy and Mercurio, 2018). The activated YAP/TAZ sustains Rho-GTPase activity via a positive feedback loop, while changes to the cytoskeletal dynamics facilitate both vascular growth and remodeling of ECs (Wang X. et al., 2017; Elaimy and Mercurio, 2018). Sakabe et al. (2017) found that YAP regulates the activity of the small GTPase CDC42, the deletion of which leads to severe defects in endothelial migration and angiogenesis. Knockdown of YAP/TAZ alters the cellular distribution of VEGFR2 due to defective trafficking from the Golgi apparatus to the plasma membrane (Elaimy and Mercurio, 2018). Xu et al. (2019) reported that TAZ expression was correlated with vascular endothelial growth factor receptor 2 (VEGFR2) immunoreactivity of ECs, and also with blood vessel density in a tumor (astrocytoma) model.

Besides the VEGF signaling axis, YAP regulates angiogenesis via the peroxisome proliferator-activated receptor gamma co-activator 1-alpha (PGC1α) signaling pathway that controls glucose metabolism within the mitochondria of ECs (Mammoto et al., 2018). Mammoto et al. (2018) showed that PGC1α knockdown inhibits YAP-induced EC sprouting *in vitro* and vascular morphogenesis within fibrin gels subcutaneously implanted into mice, whereas overexpression of PGC1α had the reverse effect. Hence, YAP-TEAD1 signaling induces mitochondrial biogenesis in ECs and stimulates angiogenesis through PGC1α. YAP also regulates angiogenesis via the STAT3 signaling pathway. Du et al. (2017) reported that YAP induces increased secretion of IL11 and IL15 from cancer-associated fibroblasts, and these cytokines in turn activate STAT3 signaling in HUVECs, promoting tubule formation and sprouting angiogenesis of these cells. Besides these canonical signaling pathways, YAP also regulates angiogenesis in tumor models via miRNAs such as miR-205 (Du et al., 2017) and miR-126-5p (Sun et al., 2019), as well as non-coding RNAs such as MALAT1 (Sun et al., 2019).

### Role of YAP/TAZ in Hepatic Differentiation and Liver Regeneration

The functions of the mammalian liver include detoxification and production of bile acids that facilitate the digestive process. The major cell lineages of the liver are hepatocytes that secrete bile acids, and biliary epithelial cells (cholangiocytes) that line the biliary ducts transporting bile acids into the gall bladder, and are responsible for modifying the bile secretions of hepatocytes. There is also a resident pool of adult mesenchymal stem cells within the post-natal liver, referred to as hepatic stellae cells, which contribute to liver regeneration upon disease or injury.

Studies on the spatiotemporal pattern of YAP expression during embryonic liver development (Du et al., 2018) and early post-natal development (Zhang K. et al., 2016) suggest that YAP activation is required for the initial proliferation of hepatoblasts, while its downregulation is required for subsequent hepatoblast differentiation and maturation. Biliary epithelial cells display consistently higher levels of YAP expression, as compared to hepatoblasts and hepatocytes, during embryonic development and early post-natal life (Zhang K. et al., 2016). *In vitro* studies have confirmed the *in vivo* findings on the role of YAP/TAZ in hepatic proliferation, differentiation and maturation. Yi et al. (2016) utilized an *in vitro* hepatocyte differentiation assay to show that YAP activity decreases, whereas Hippo pathway kinase activities (LATS1/2) increase, upon hepatic differentiation. Yamamoto et al. (2018) reported that cell-aggregate formation results in actin reorganization and intercellular adhesion, which in turn rapidly induces growth arrest and maturation of induced hepatocyte-like (iHep) cells through activation of Hippo signaling. The resulting inactivation of YAP induces upregulation of Hnf1α expression, which acts as a key transcription factor that enhances hepatocyte-specific gene expression within cell aggregates, thereby promoting functional maturation of iHep cells. Conversely, Lee et al. (2016) showed that YAP activation suppresses hepatoblast-to-hepatocyte differentiation by repressing Hnf4α expression.

YAP/TAZ activation is required for the mobilization and proliferation of hepatic stellae cells, the endogenous resident adult stem cell pool within the liver that contributes to regeneration following disease or injury. Konishi et al. (2018) observed inactivation of the Hippo signaling pathway and concomitant YAP activation specifically in hepatic stellae cells, upon mobilization and proliferation of these cells in response to ischemia-reperfusion injury of mouse liver. Conversely, treatment of mice with verteporfin, a potent YAP/TAZ inhibitor, drastically reduces hepatic stellae cell proliferation and the regenerative capacity of the liver after ischemia-reperfusion injury. It can be concluded that in normal healthy livers, the Hippo signaling pathway is responsible for maintaining hepatic stellae cells in a quiescent state through YAP inactivation.

Although hepatic stellae cell activation is essential for liver regeneration, it should be noted that excessive YAP activation in stellae cells, particularly during chronic liver damage, results in fibrosis and ultimately cirrhosis of the liver (Mannaerts et al., 2015). Indeed, pharmacological inhibition or knockdown of YAP mitigated liver fibrosis in mice by impeding hepatic stellate cell activation (Mannaerts et al., 2015). Hence, in the quest for new drugs to impede liver fibrosis and cirrhosis, it may be useful to search for other non-Hippo related signaling pathways that regulate YAP activation in hepatic stellae cells. Signaling pathways that have so far been implicated include the TGF-β1 signaling pathway (Lee et al., 2016; Yu et al., 2019), the Wnt/β-catenin signaling pathways (Yu et al., 2019), the Notch signaling pathway (Tharehalli et al., 2018) and the Hedgehog signaling pathway (Swiderska-Syn et al., 2016; Du et al., 2018). In addition, Zhang K. et al. (2016) demonstrated that uptake of omega-3 polyunsaturated fatty acids by mice ameliorates liver fibrosis by inhibiting hepatic stellae cell activation and proliferation through promotion of YAP/TAZ degradation.

### Role of YAP/TAZ in Myogenic Differentiation and Skeletal Muscle Regeneration

Postnatal skeletal muscle tissue contains a resident pool of adult stem cells known as satellite cells, which are normally quiescent. However, upon muscle injury, muscle satellite cells are activated to proliferate and generate myoblasts, which in turn give rise to terminally differentiated myotubes of muscle fibers. Judson et al. (2012) revealed that YAP plays a crucial role in the cell fate determination of muscle satellite cells. Satellite cell activation into highly proliferative myoblasts is associated with increased YAP nuclear translocation. The binding of YAP to TEAD transcription factors within satellite cells was shown to co-activate MCAT elements that are enriched in the proximal promoters of YAP-responsive genes, such as BMP4, CD34, and Myf6 (Mrf4) (Judson et al., 2012). Li and Fan (2017) showed that CREB (cAMP response element-binding protein), MPP7 and AMOT are required for nuclear translocation of YAP during satellite cell activation, as well as for maintenance of the proliferative state in myoblasts. Inhibition of CREB activity in satellite cells causes these cells to remain quiescent even upon injury, and they become unable to transit into a proliferative state for expansion and self-renewal.

There is evidence that YAP activation in satellite cells and myoblasts involve mechanotransduction. Goodman et al. (2015) reported that in skeletal muscle tissues, mechanical overload promotes an increase in YAP expression, which in turn induces skeletal muscle hypertrophy via a rapamycin complex 1 (mTORC1)-dependent mechanism. Brown et al. (2018) utilized a photo-crosslinkable hydrogel model to probe myoblast mechanotransduction in three dimensions, and demonstrated that increased matrix stiffness decreases cell spreading and reduces nuclear localization of YAP, whereas a reduction of matrix stiffness had the opposite effect. Stearns-Reider et al. (2017) implicated mechanotransduction in the age-related decline of skeletal muscle regeneration. It was demonstrated that aged muscle has an increasingly stiff matrix microenvironment, which results in increased nuclear translocation of YAP. This in turn inhibits the further differentiation and maturation of myoblasts into myotubes.

Besides its role in satellite cell activation and myoblast proliferation, YAP activity also plays a role during the early phase of myoblast differentiation, by regulating mitochondrial structural remodeling. Huang et al. (2018) demonstrated that during early myoblast differentiation, YAP upregulates the expression of dynamin-related protein 1 (Drp1), leading to an increased number of mitochondrial fission events. Downregulation of YAP inhibits myoblast differentiation by decreasing expression of dynamin-related protein 1 (Drp1), resulting in elongated mitochondria, fused mitochondrial networks, and collapsed mitochondrial membrane potential. Chen et al. (2017) implicated the MEK5-ERK5 pathway in early myoblast differentiation mediated by YAP.

Nevertheless, further differentiation and maturation into myotubes require subsequent deactivation of YAP through phosphorylation. Watt et al. (2010) demonstrated that during the later phase of myoblast differentiation into myotubes, phosphorylation of YAP increases almost 20-fold as YAP translocates from the nucleus to the cytosol. In addition, myoblast differentiation into myotubes was inhibited by overexpression of mutant YAP that cannot be phosphorylated. The role of YAP deactivation in the further differentiation and maturation of myoblasts into myotubes was further validated by Vita et al. (2018), who showed that the lack of skeletal muscle development and regeneration observed in Duchenne muscular dystrophy is associated with inactivation of the Hippo signaling pathway and increased YAP nuclear translocation.

Interestingly, although TAZ, like YAP, plays a role in the proliferation of myoblasts, the two proteins have divergent functions in myoblast differentiation. Sun et al. (2017) demonstrated that in the later stages of myogenesis, TAZ promotes myoblast differentiation and maturation into myotubes, in contrast to the inhibitory effect of YAP. TAZ also operates through TEAD4 to enhance myogenic differentiation (Sun et al., 2017). Feng et al. (2019) showed that the dual function of VGLL4 promotes muscle regeneration via modulation of YAP/TAZ activity. By repressing YAP activity, VGLL4 enhances the further differentiation and maturation of myoblasts into myotubes. At the same time, it serves as a co-activator of TEAD4, which is targeted by TAZ during the later phase of myogenesis to activate myogenin (MyoG) expression (Sun et al., 2017; Feng et al., 2019).

Hence, in conclusion, YAP/TAZ activity is required for satellite cell activation and proliferation, as well as for the early phase of myoblast differentiation. In contrast, the roles of YAP and TAZ diverge during the later phase of myoblast differentiation and maturation into myotubes, which require TAZ activation and YAP deactivation.

### Role of YAP/TAZ in Cardiomyogenesis and Heart Regeneration

The role of YAP/TAZ in cardiomyogenesis was first revealed by studies on fetal heart development. Fetal cardiomyocytes undergo extensive proliferation that ends abruptly after birth, and these changes are strongly correlated with YAP activation and deactivation, respectively (Del Re et al., 2013; Xin et al., 2013; Lin et al., 2014, 2015; Mosqueira et al., 2014; Cho et al., 2017; Estarás et al., 2017; Hou et al., 2017; Khalafalla et al., 2017; Mills et al., 2017; Mochizuki et al., 2017; Ragni et al., 2017; Artap et al., 2018; Wang X. et al., 2018; Ito et al., 2019; Torrini et al., 2019). Postnatal heart tissue growth is driven primarily by cardiomyocyte hypertrophy (increase in cell size and deposition of extracellular matrix), rather than increase in cell number (von Gise et al., 2012; Xin et al., 2013; Artap et al., 2018). A gain and loss of function study by von Gise et al. (2012) demonstrated that YAP activation is required for cardiomyocyte proliferation during fetal heart growth, but not for cardiomyocyte hypertrophy during postnatal heart growth. YAP activation in postnatal cardiomyocytes stimulates proliferation (von Gise et al., 2012). Xin et al. (2013) showed that YAP promotes the proliferation of embryonic cardiomyocytes through activation of the insulin-like growth factor and Wnt signaling pathways, while Artap et al. (2018) reported that YAP/TAZ may promote myocardial growth within the fetus via paracrine secretion of neuregulin.

Several studies have reported increased YAP/TAZ activation in diseased hearts, suggesting a role in heart repair and regeneration. Hou et al. (2017) reported increased activation of YAP/TAZ signaling in ischemic heart disease and dilated cardiomyopathy. Interestingly, it was observed that both human and mouse diseased hearts initially express more TAZ than YAP at the mRNA and protein levels, though any subsequent increases in the expression of these two homologs in diseased hearts are proportional and the YAP/TAZ ratio remains unchanged. Del Re et al. (2013) found that YAP promotes cardiomyocyte survival and growth after myocardial infarction, and showed that heterozygous deletion of YAP significantly decreases cardiomyocyte proliferation and exacerbates injury in response to chronic myocardial infarction. Lin et al. (2014) utilized a cardiac-specific, inducible expression system to demonstrate that YAP activation after myocardial infarction preserves cardiac function and enhances the survival of cardiomyocytes, while Xin et al. (2013) showed that transgenic expression of a constitutively active form of YAP in adult heart stimulates cardiac regeneration and improves contractility after myocardial infarction.

Various signaling pathways have been implicated in YAP-induced cardiac cell proliferation. Wang X. et al. (2018) reported that treatment of neonatal cardiomyocytes with poly (I:C), a Toll-like receptor 3 (TLR3) ligand, significantly enhances glycolytic metabolism, which triggers YAP activation and subsequent cell proliferation. Conversely, 2-deoxyglucose (2-DG), a glycolysis inhibitor, blocks proliferation. Wang X. et al. (2018) also showed that YAP activation upregulates miR-152, which represses the expression of cell cycle inhibitory proteins P27kip1 and DNMT1, thereby promoting cardiomyocyte proliferation. Torrini et al. (2019) identified miR-199a-3p as having pro-proliferative effects on cardiomyocytes through direct targeting of mRNAs of two proteins involved in YAP degradation, the upstream YAP inhibitory kinase TAOK1, and the E3 ubiquitin ligase β-TrCP. Khalafalla et al. (2017) reported that stimulation of the P2Y2 nucleotide receptor promotes cardiac progenitor cell proliferation through YAP activation. Lin et al. (2015) linked the phosphoinositol-3-kinase-Akt and Hippo-YAP signaling pathways in the regulation of cardiomyocyte proliferation and survival, and identified the p110β catalytic subunit of phosphoinositol-3-kinase as the nexus between the two pathways. *In vitro* stimuli that trigger cardiomyocyte proliferation through YAP activation include substrate rigidity and nanostructure (Mosqueira et al., 2014), and treatment with the small-molecular drug TT-10 (C11H10FN3OS2) (Ito et al., 2019), but the underlying molecular mechanisms remain poorly characterized (Mosqueira et al., 2014; Ito et al., 2019).

As in the case of other lineages, after an initial proliferation phase triggered by YAP activation, there is a need for subsequent YAP deactivation for further differentiation and maturation of cardiac stem/progenitor cells to terminally differentiated cardiomyocytes. This is usually associated with the restriction of heart growth at birth (Ragni et al., 2017). Utilizing a cardiac organoid model, Mills et al. (2017) showed that YAP deactivation is associated with a switch to fatty acid metabolism that in turn inhibits proliferation and promotes cardiomyocyte maturation. Mochizuki et al. (2017) reported that YAP deactivation leads to downregulation of polo-like kinase 2, which in turn enables cardiac progenitors to switch from the proliferative to the terminal differentiation phase.

Interestingly, some studies have found that YAP deactivation also facilitates the cardiac lineage commitment of other stem cell types (Cho et al., 2017; Estarás et al., 2017). Using human embryonic stem cells, Estarás et al. (2017) showed that YAP deactivation facilitates activin-induced Wnt3 expression, as well as stabilizing β-catenin, which then synergizes with activin-induced SMAD signaling to activate a subset of mesodermal genes required to form the cardiac mesoderm. Cho et al. (2017) demonstrated that treatment of mesenchymal stem cells with apicidin deactivates YAP, leading to downregulation of miR-130a expression. This in turn induces expression of cardiac markers, such as GATA4, Nkx2.5, and cardiac troponin I, in MSCs.

### Role of YAP/TAZ in Epidermal/Keratinocyte Differentiation and Skin Regeneration

The skin is the largest organ in the human body, and consists of the outer epidermis and inner dermis, separated by a basement membrane. The outer epidermis forms the exterior covering of the body, and is constantly being subjected to a barrage of environmental insults and physical injuries. Epidermal healing and regeneration are mediated primarily by a resident pool of adult stem cells that are referred to as epidermal stem cells or keratinocyte stem cells. These cells are located in the basal layer of the epidermis, attached to the basement membrane. Beverdam et al. (2013) showed that YAP functions as a molecular switch of epidermal stem/progenitor cell activation in the epidermis, and that the C-terminus of the YAP protein regulates the balance between stem/progenitor cell proliferation and differentiation. The role of YAP/TAZ in skin regeneration was confirmed by Lee et al. (2014), who found that knockdown of YAP/TAZ by small interfering RNA (siRNA) impairs the healing process in full-thickness skin wounds.

As mentioned in section “Role of YAP/TAZ in Stem Cell Self-Renewal and Maintenance of Stem Cell Phenotype,” mechanosensing of the extracellular matrix involves the regulation of YAP/TAZ activity via complex interactions of FAs, interconnected actin fibers and RhoA with various components of the Hippo signaling pathway. Through these mechanistic pathways in epidermal stem cells, YAP/TAZ act as sensors of mechanical forces and skin tissue damage, as well as switches between quiescence, proliferation and differentiation (Elbediwy and Thompson, 2018). Totaro et al. (2017) reported that mechano-activation of YAP/TAZ promotes epidermal stemness via inhibition of Notch signaling, which promotes epidermal differentiation. Conversely, YAP/TAZ inhibition by weak mechanical forces induces Notch signaling and differentiation, with loss of stem cell characteristics (Totaro et al., 2017).

Besides mechanotransduction, other upstream signaling mechanisms regulate YAP/TAZ-induced proliferation of epidermal stem cells. Flores and Halder (2011) identified the AJ component α-catenin as a regulator of epidermal stem cell quiescence in healthy skin tissues through its inhibition of YAP activation. Schlegelmilch et al. (2011) found that α-catenin controls YAP activity and phosphorylation by modulating the interaction with 14-3-3 and PP2A phosphatase. Elbediwy et al. (2016) reported that YAP/TAZ activation and triggering of proliferation in epidermal stem cells are mediated by integrin-Src signaling arising from cellular contact with the basal layer extracellular matrix, and that YAP/TAZ is subsequently deactivated in differentiating daughter cells upon loss of cellular contact with the basement membrane. Zhang B. et al. (2016) demonstrated that exosome-delivered 14-3-3ζ protein promotes the phosphorylation of YAP and its subsequent translocation to the cytosol by mediating its binding to p-LATS, a component of the Hippo signaling pathway. This in turn restricts excessive proliferation of epidermal stem cells and enhances collagen deposition during skin healing (Zhang B. et al., 2016). Walko et al. (2017) reported that epidermal proliferation involves YAP interaction with the WW-binding protein 2 (WBP2) co-factor, which results in enhanced YAP/TEAD-mediated gene transcription.

Downstream target genes activated by YAP/TEAD that are involved in the proliferation of epidermal stem/progenitor cells include Plau and TGF-βr3 (Corley et al., 2018), and Cyr61 (Zhang et al., 2011), Hoxa1 and Hoxc13 (Liu et al., 2015). Mendoza-Reinoso and Beverdam (2018) reported that WNT16 is upregulated in response to YAP activation in keratinocytes, resulting in promotion of keratinocyte proliferation via the canonical WNT16/β-catenin signaling pathway. In accordance with this, Akladios et al. (2017b) found that a YAP-induced increase of β-catenin expression is essential for proliferation of basal keratinocytes. Other YAP/TAZ-activated downstream signaling pathways that modulate proliferation of epidermal stem/progenitor cells include the Notch (Akladios et al., 2017a) and GLI2/Hedgehog signaling pathways (Akladios et al., 2017a).

### Role of YAP/TAZ in Intestinal Epithelium Differentiation and Regeneration

The inner lining (luminal surface) of the gastrointestinal tract consists of a single-cell layer of simple columnar epithelial cells, which is known as the intestinal epithelium. This serves two major functions: (i) absorption of nutrients and other useful substances into the body, and (ii) preventing the entry of toxic and harmful substances. Endogenous adult stem cells residing within the intestinal epithelium are called intestinal stem cells, and play a crucial role in the homeostasis and regeneration of the intestinal epithelium, which is a rapidly cycling tissue that renews every 4 to 5 days under normal conditions (Zhang and Huang, 2013).

As in most other tissues, YAP/TAZ are involved in the proliferation and differentiation of intestinal stem cells (Le Bouteiller and Jensen, 2015). Imajo et al. (2015) reported that, although high levels of YAP/TAZ activation promote proliferation and inhibit differentiation of intestinal stem cells, more moderate levels of YAP/TAZ activation promote both proliferation and differentiation of these cells into mucus-secreting goblet cells. TEADs and Klf4 were identified as the partner transcription factors of YAP/TAZ in the proliferation and differentiation processes, respectively (Imajo et al., 2015).

As in the cases of other cell lineages, mechanosensing by YAP/TAZ also serves as a switch between quiescence, proliferation and differentiation in intestinal stem cells. Gjorevski et al. (2016) reported that fibronectin-based adhesion and high matrix stiffness significantly enhanced intestinal stem cell proliferation and expansion via a YAP-dependent mechanism, whereas differentiation is enhanced by laminin-based adhesion on a soft matrix. Liu et al. (2017) suggested that RhoA plays a key role in the upstream regulation of YAP-based mechanosensing by intestinal stem cells. Other upstream regulators of YAP activity in intestinal stem cells are also essential for maintaining the fine balance between quiescence, proliferation and differentiation within the intestinal epithelium, which if disrupted, leads to tumorigenesis (Zhou et al., 2011; Llado et al., 2015). These regulators include protein kinase C ζ (Llado et al., 2015) and MST1/2 (Zhou et al., 2011).

Various signaling pathways regulating cell proliferation are known to be downstream targets of YAP/TAZ activation within intestinal stem cells, including the epiregulin/epidermal growth factor receptor (EGFr), β-catenin and Wnt signaling pathways (Zhou et al., 2011; Barry et al., 2013; Gregorieff et al., 2015; Llado et al., 2015; Liu et al., 2017). While the epiregulin/EGFr and β-catenin signaling pathways are both activated by YAP to promote cell proliferation (Zhou et al., 2011; Llado et al., 2015; Liu et al., 2017), the Wnt signaling pathway, in contrast, is suppressed by YAP (Barry et al., 2013; Gregorieff et al., 2015). With intestinal stem cells, activation of Wnt signaling promotes cell proliferation, as well as increased differentiation into Paneth cells of small intestinal crypts, which play a pivotal role in innate immune defense of the gut through the secretion of anti-microbial proteins (Barry et al., 2013; Gregorieff et al., 2015). Hence, YAP suppression of the Wnt signaling pathway within intestinal stem cells serves not only as a counterbalance against excessive cell proliferation, but also to inhibit differentiation into Paneth cells and reduce the formation of intestinal crypts (Barry et al., 2013; Gregorieff et al., 2015).

Interestingly, Yui et al. (2018) proposed that YAP/TAZ also have a role in transient reprogramming of the intestinal epithelium into a primitive state during the regeneration process, which is characterized by *de novo* expression of fetal markers, as well as suppression of markers for adult stem cells and differentiated cells. This can be recapitulated *in vitro* with a collagen 3D matrix supplemented with Wnt ligands, which serve to sustain endogenous YAP/TAZ activation and induce reprogramming of cell fate (Yui et al., 2018).

## Conclusion and Future Outlook

In recent years, there has been rapid increase in our knowledge of the signaling mechanisms by which YAP/TAZ regulate the development, homeostasis and regeneration of various tissue/organ lineages within the human body. Despite the great interest in the roles of YAP/TAZ in cancer and tumorigenesis, as much attention, if not more, has been focused on how YAP/TAZ maintains the delicate balance between quiescence, self-renewal, proliferation and differentiation of endogenous adult stem cells in various different tissue types during the processes of tissue regeneration and healing. In fact, we cannot really separate these two interrelated fields, given the widely accepted idea that cancers and tumors originate from aberrant adult stem cells (Sell, 2010).

New insights into YAP/TAZ can potentially impact the stem cell and regenerative medicine field in many ways. Firstly, we can exploit our knowledge of the key roles of YAP/TAZ in maintaining stem cell proliferation and self-renewal during the regeneration process, to scale-up cell culture in order to obtain sufficient numbers of cells for therapeutic applications. Assaying YAP/TAZ activity by means of high-throughput screening technology can identify potential drug leads with the ability to enhance the proliferative and self-renewal capacity of various kinds of adult stem cell lineages.

Secondly, a better understanding of the role of YAP/TAZ in cell lineage fate determination would lead to improved *in vitro* culture systems and protocols for differentiating adult, embryonic and induced pluripotent stem cells into specific lineages. More precise control of cell lineage determination would be advantageous for therapeutic applications in tissue engineering and regenerative medicine, as well as for non-therapeutic applications in pharmacology and toxicology screening assays. For example, based on existing knowledge that the level of YAP/TAZ activity in MSCs is crucial for specification into osteogenic, chondrogenic or adipogenic lineages, it may be possible to design improved *in vitro* culture systems for enhancing differentiation into a pre-selected lineage through more precise control of YAP/TAZ activity in the cells. This might be achieved by modifying the biomechanical and topographical properties of the substrata, or supplementation of small molecules or growth factors that modulate YAP/TAZ activity.

Thirdly, we may exploit our new knowledge of YAP/TAZ signaling mechanisms to optimize the therapeutic properties of newly developed biomaterials. By performing YAP/TAZ assays on relevant cell lineages cultured on newly developed biomaterials *in vitro*, we may be able to fine-tune various biomechanical properties such as stiffness (Dupont et al., 2011; Nardone et al., 2017; Pardo-Pastor et al., 2018) and topography (Yang W. et al., 2016; Zhang Y. et al., 2016; Arslan et al., 2017; Hwang et al., 2017; Qian et al., 2017), enabling us to optimize the healing and regeneration processes at specific tissue/organ sites with implanted biomaterials.

Lastly, a newly emerging research frontier on how YAP/TAZ modulate stem cell function, are their novel roles in effecting epigenetic modifications to chromatin structure (Hillmer and Link, 2019) and post-transcriptional miRNA processing (Chaulk et al., 2014; Mori et al., 2014). Epigenetic modifications mediated by YAP/TAZ are effected by their association with chromatin-remodeling complexes proteins such as Nucleosome Remodeling and Deacetylase (NuRD), Switch/sucrose non-fermentable (SWI/SNF), Ncoa6, Mediator, and GAGA, which in turn influence the accessibility and activity of various target genes via alterations to chromatin structure (Hillmer and Link, 2019). Post-transcriptional processing of miRNA is known to be regulated by YAP/TAZ via their binding interactions with p72 (DDX17) that regulates Microprocessor activity (Mori et al., 2014), as well as by modulation of Dicer activity through the LIN28/Let-7 axis (Chaulk et al., 2014). Both these regulatory mechanisms can potentially exert profound effects on stem cell phenotype and function. Nevertheless to date, there has not yet been any comprehensive studies on these YAP/TAZ-associated regulatory mechanisms within the stem cell and regenerative medicine field.

## Author Contributions

BH and XZ contributed equally to writing most of the manuscript sections. DA prepared the figures and figure legends. YB, XL, and YW wrote some of the manuscript sections. MF and XD provided supervision and funding. All authors contributed to the article and approved the submitted version.

## Conflict of Interest

The authors declare that the research was conducted in the absence of any commercial or financial relationships that could be construed as a potential conflict of interest.
